# Identification of a novel PAX6 mutation in a Chinese family with aniridia

**DOI:** 10.1186/s12886-018-1009-6

**Published:** 2019-01-08

**Authors:** Jing-Jing Qiu, Qian Zhang, Zi-xin Geng, Min Liu, Zi-lin Zhong, Jian-jun Chen, Fei Liu

**Affiliations:** 1 Department of Ophthalmology, the Second Affiliated Hospital of Nanchang University, No.1 Minde Road, Donghu District, Nanchang, 330006 China; 2 Department of Ophthalmology of Shanghai Tenth People’s Hospital, and Tongji Eye Institute, Tongji University School of Medicine, Shanghai, 200092 China; 3 Department of Medical Genetics Tongji University School of Medicine, Shanghai, 200092 China

**Keywords:** Aniridia, Autosomal dominant inheritance, *PAX6* gene, Mutation

## Abstract

**Background:**

This study aims to investigate the clinical characterization and causative genetic defect of a four-generation Chinese family with autosomal dominant aniridia.

**Methods:**

The recruited family members underwent comprehensive routine and ophthalmic examinations, and Sanger sequencing was performed to screen the mutation in *PAX6*.

**Results:**

A novel heterozygous *PAX6* deletion c.435_445delTAGCGAAAAGC (p.Ser146ThrfsX9) in exon 7 was identified in all affected individuals, but this was absent in any of the unaffected family members and in the 200 unrelated controls.

**Conclusion:**

A novel deletion in the *PAX6* gene was identified in a Chinese family associated with aniridia, which expands the spectrum of the PAX6 mutation and its associated phenotype.

## Background

Aniridia is a congenital ocular abnormality, in which the most characteristic feature is iris hypoplasia associated with the gene mutation of *paired box-6* (*PAX6*) in most cases. In order to investigate the clinical characterization and causative genetic defect in this four-generation Chinese family with autosomal dominant aniridia, the recruited family members underwent comprehensive routine and ophthalmic examinations, and Sanger sequencing was performed to screen the mutation in *PAX6*. A novel heterozygous *PAX6* deletion c.435_445delTAGCGAAAAGC (p.Ser146ThrfsX9) in exon 7 was identified in all affected individuals, but this was absent in any of the unaffected family members and in the 200 unrelated controls. Hence, it was concluded that a novel deletion in the *PAX6* gene was identified in a Chinese family associated with aniridia, which expands the spectrum of the *PAX6* mutation and its associated phenotype.

Aniridia is a serious congenital abnormality in the iris, which is usually bilateral. The main clinical features include a partially or completely missing iris, and decreased vision and nystagmus in some patients. Aniridia can be accompanied by other ocular diseases, such as cataracts, clouding of the cornea, glaucoma, macular abnormality, optic nerve hypoplasia and so on. Approximately 13% of aniridia cases occur in WAGR (Wilms tumor, aniridia, genitourinary disorders, and mental Retardation) syndrome [1]. Furthermore, the prevalence rate of congenital aniridia is approximately 1:64,000–1:96,000 worldwide [2], and approximately 1:100,000 in China [3]. Moreover, two-thirds of aniridia cases have an obvious hereditary history, and are almost in the pattern of autosomal dominant inheritance, while the remaining cases refer to sporadic cases [4–6].

Approximately 80% congenital aniridia cases are caused by the gene mutation of human *paired box-6* (*PAX6*) [7–9]. The Human *PAX6* Allelic Variant Database (http://lsdb.hgu.mrc.ac.uk/home.php?select_db=PAX6) has recorded over 400 *PAX6* mutations, to date [10]. Most of these mutations are frameshift mutations, splicing site mutations, or nonsense mutations, which have been considered to produce truncated or nonsense transcripts, leading to haploinsufficiency associated with ocular abnormal signs, while other mutations were missense [11–13]. The PAX6 protein is a functionally conserved transcription factor that can recognize and bind to a specific DNA sequence to regulate the expression of target genes, and is a transcription activation function that can activate downstream genes regulated by the PAX6 protein [14, 15].

In the present study, all exons and flanking regions of *PAX6* were screened in a Chinese family with aniridia, and a novel heterozygous deletion was detected.

## Methods

### Participants and clinical data

The Institutional Review Board (IRB) of Tongji Eye Institute of Tongji University, School of Medicine (Shanghai, China), and the Second Affiliated Hospital of Nanchang University (Nanchang, Jiangxi Province, China) approved the present study. All participating family members provided an informed written consent, and were endorsed by their respective IRB. The whole procedure of the present study adhered to the tenets of the Declaration of Helsinki.

Family 12,002, a four-generation family with aniridia, was recruited for the present study. This family included four affected and eight unaffected members. Routine and ophthalmological examinations (including vision, cornea, iris, lens, fundus, intraocular pressure, etc.) were performed on the affected individuals, as well as on the unaffected family members. In addition, 200 ethnically matched healthy individuals with no direct or collateral ties and no ocular and systemic underlying diseases were recruited.

### Mutation analysis

The peripheral venous blood genomic DNA of the family and healthy members of the family was extracted by phenol-chloroform extraction. The experimental procedures were conducted according to the protocol of the RelaxGene Blood DNA extraction kits (TianGen, Beijing, China). All 14 exons of PAX6 plus the flanking regions were amplified by polymerase chain reaction (PCR). The primers for the amplification were designed by the web-based version of the Primer 3 program. The PCR amplification was performed using a BIO-RAD T100 ThermalCycler PCR amplifier. The primer sequences and amplification conditions are presented in Table 1. Sanger sequencing was performed for all members of Family 12,002 with available DNA samples using an ABI3730 Automated Sequencer (PE Biosystems, Foster City, CA, USA). The sequencing results were sequenced using the DNASar software to screen for *PAX6* gene mutation sites and mutation types, and compare these with *PAX6* gene sequences from another cohort of 200 unrelated ethnically-matched controls.

**Table 1:** Primers used for amplification and sequence analysis of human PAX6

Exon	Forward Sequence 5′-3′	Reverse Sequence 5′-3′
EXON04	AGATCGCCCCAAGAGGTTG	ATCGAGAAGAGCCAAGCAAAC
EXON05	GGTGGTCCTGTTGTCCTTTAT	GGGGTCCATAATTAGCATCGT
EXON06–07	AGCTCTCTACAGTAAGTTCTCA	CCCAGGTACAAAGGAGACAAA
EXON08	TCCGCCCAATTCTCTATCCAA	TACACAACCCTCACATTCCCA
EXON09	GGTGAGGCTGTCGGGATATAA	TCTTTGTACTGAAGATGTGGCA
EXON10–11	TAACTTGGTTCTGGTGGGAAA	CGGAGCAAACAGGTTTAAAGA
EXON11–12	TGCTAACCTGTCCCACCTG	GAAAAGCTCTCAAGGGTGCAG
EXON12–13	AGGCTTGATACATAGGCAGCT	GGACAAGGAAAGCAAGGAGTT
EXON14	TGTATTCCATGTCTGTTTCTCA	GGTACAATACAGGACACAATTG

Note: All primers were amplified using a touchdown protocol beginning at 64 °C, decreasing

by 0.5 °C each cycle, until finishing at a final annealing temperature of 57°°C

## Results

### Clinical findings

**Fig. 1:**
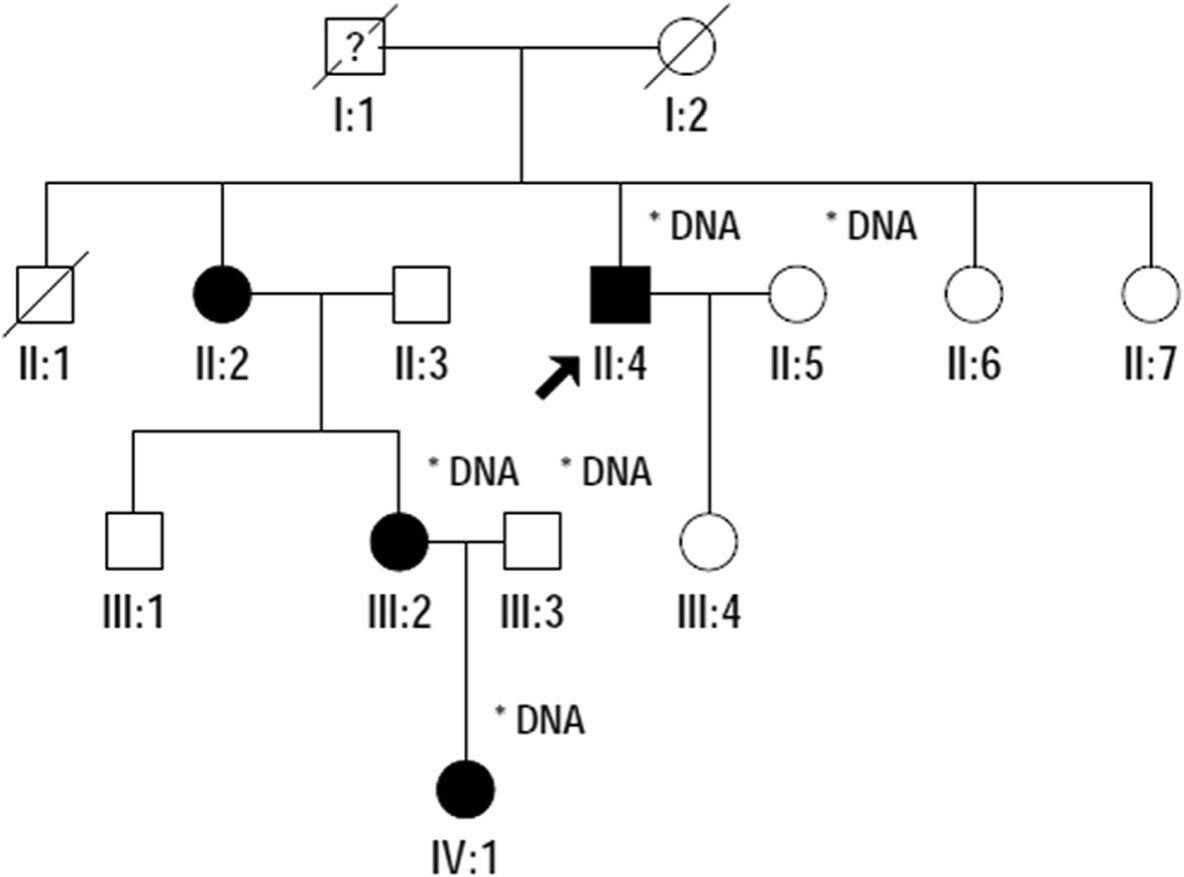
Pedigree of Family 12,002 with aniridia. Squares indicate males, and circles, females. The arrow points out the proband. Empty symbols and filled symbols show normal individuals and affected patients, respectively

**Fig. 2:**
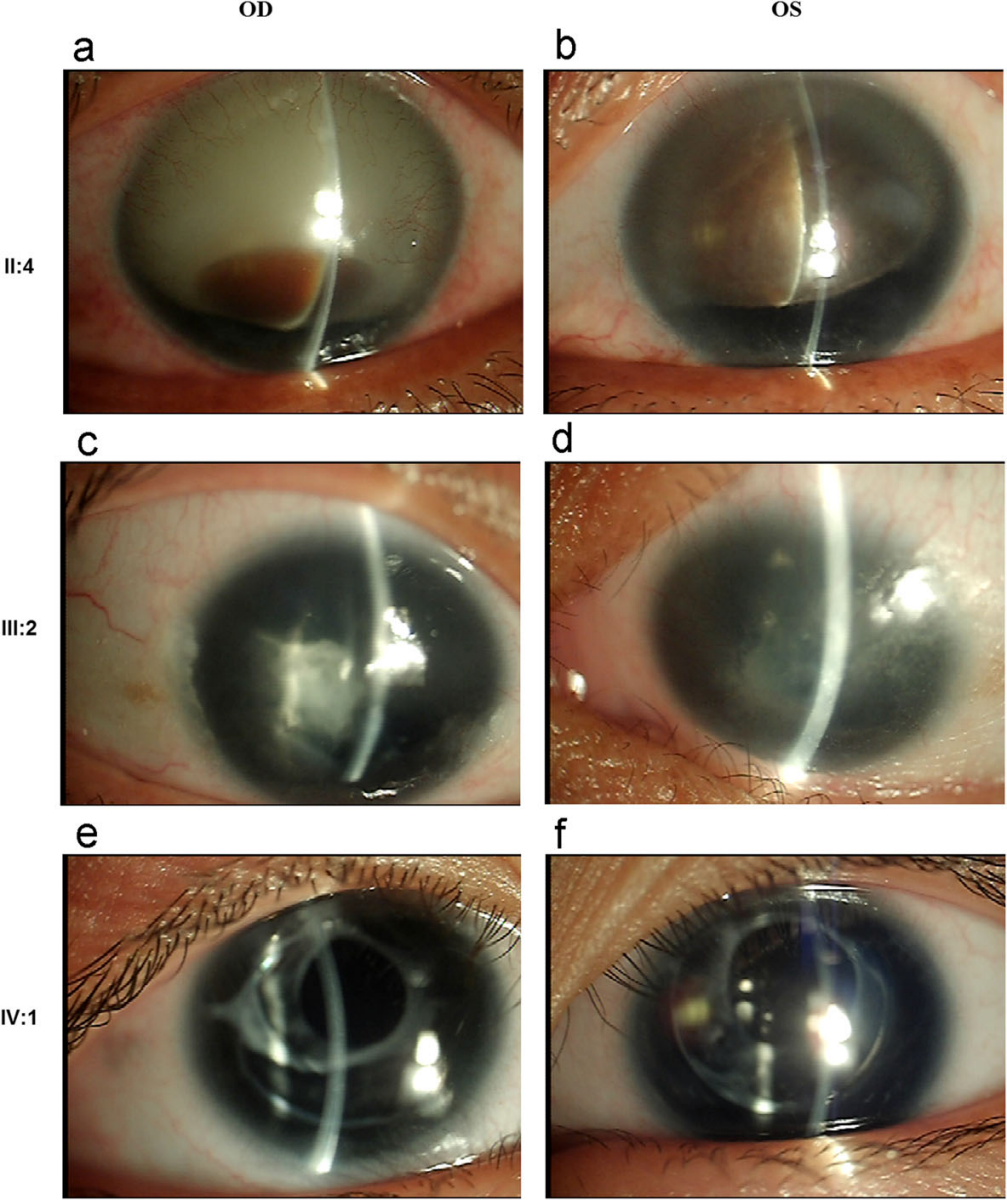
Clinical features of the affected in Family 12,002. Slit-lamp photographs of the patients, proband (**a**, **b**), the proband’s niece (**c**, **d**) and her son (**e**, **f**). OD stands for right eye, OS, left eye

Family 12,002 is a four-generation family, and aniridia continuously occurred in the II, III and IV generations. This was consistent with the features of an autosomal dominant inheritance pattern (Fig. 1). All affected members had bilateral eye disease. The common eye abnormalities included low vision in both eyes, complete absence of the iris, and horizontal tremor in the eye. The different patient-specific eye manifestations were as follows: the proband (II:4) was a 46-year-old male with corneal haze, nystagmus, ptosis, strabismus, glaucoma, photophobia and cataract, and was lens ectopic (Fig. 2a and b); the niece of the proband (III:2) was 25 years old, has binocular cataract, and the left corneal matrix was turbid (Fig. 2c and d); her son (IV:1) had a developing cataract at five years old (Fig. 2e and f). There was no other disease or abnormalities in all the affects.

### Mutation analysis

The *PAX6* gene sequence of the proband was compared with the *PAX6* gene sequence of healthy individuals. The coding and splicing regions of *PAX6* were analyzed by bi-directional sequencing. The *PAX6* gene exon 7 was found to have a heterozygous deletion mutation in proban c.435_445 delTAGCGAAAAGC (p.Ser146ThrfsX9). This mutation encodes the linker region between PD and HD, forms a premature termination codon (PTC), and finally results in PAX6 underdosage (II:4, Fig. 3). The mutation was detected in all other affected members in the family, but not in any of the unaffected members and in the 200 unrelated controls from the same ethnic background.

**Fig. 3:**
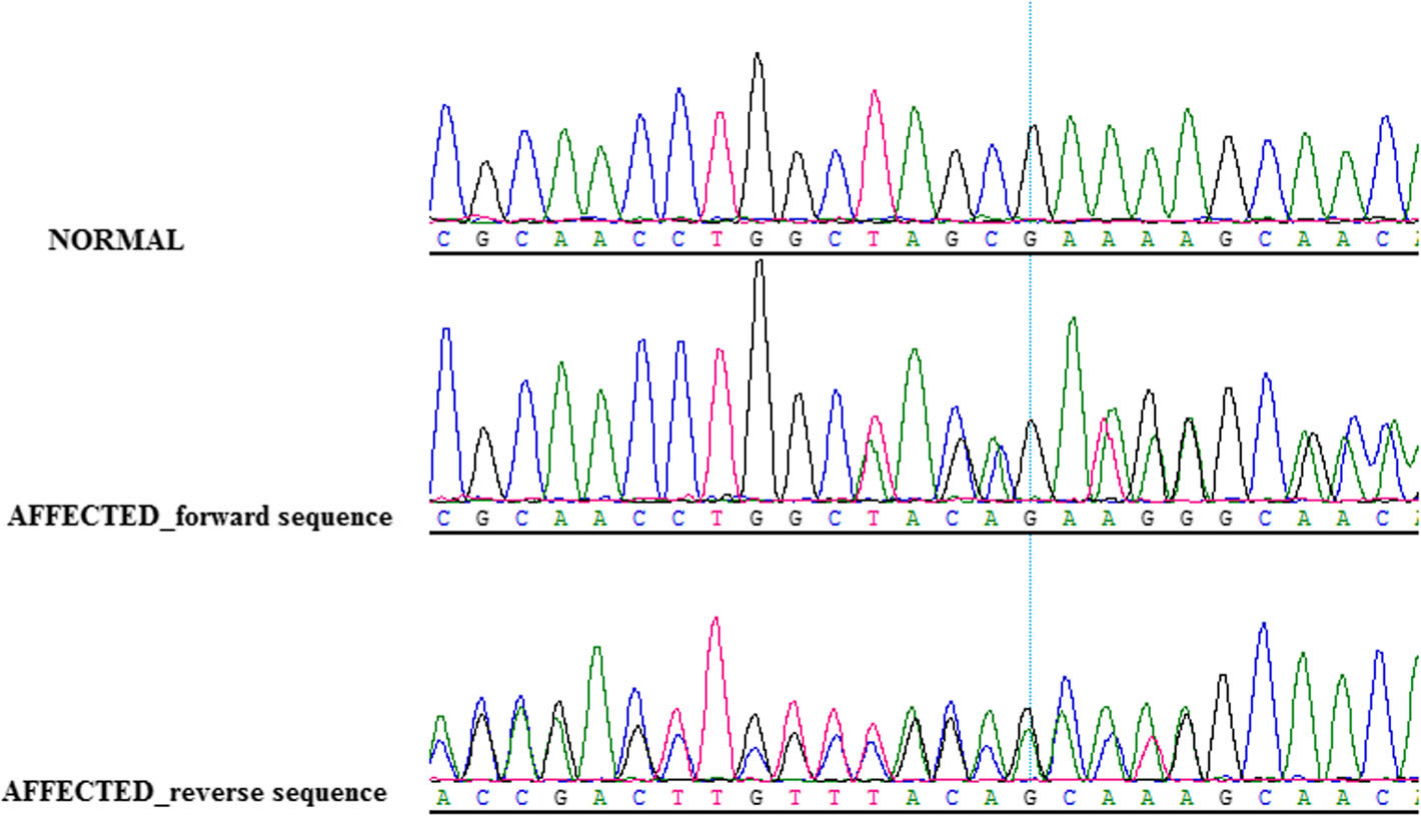
Sequence chromatograms showing the *PAX6* mutation identified in this study and wild-type form

In addition, the identified mutation has not been recorded in the databases of probably benign variations, including the database of single nucleotide polymorphisms (dbSNP), Exome Variant Server database, or the 1000 genomes project dataset. Furthermore, the mutation was absent in either the Human *PAX6* Allelic Variant Database (http://lsdb.hgu.mrc.ac.uk/home.php?select_db=PAX6), or reported literatures. These results suggest that this mutation of *PAX6* is a novel causative mutation for autosomal dominant congenital aniridia.

## Discussion

Congenital aniridia is a clinically rare hereditary eye disease. The most prominent clinical feature is the degeneration of the iris tissue, which is characterized by partial or complete loss of the iris, with or without foveal dysplasia and nystagmus. It often occurs with developmental glaucoma, congenital cataract and corneal abnormalities. The *PAX6* (paired box gene 6) gene is the most important gene that leads to congenital aniridia. At least 90% of iris-free cases are caused by heterozygous mutations in the *PAX6* gene. Furthermore, few studies have found that *FOXC1* and *PITX2* gene mutations can also lead to aniridia. [11]. *PAX6* belongs to the *Pax* gene family, and is located on human chromosome 11. Its encoded transcription factor is highly conserved during biological evolution, and participates in the embryonic development of various tissues and organs, especially in the development of the eye [10].

The clinical manifestations of aniridia are diverse. According to literature statistics, in the LOVDPAX6 gene database, 90% of disease-causing mutations lead to the aniridia phenotype, while the remaining 10% causes follicular dysplasia, Peters Syndrome and small eyeballs. [4]. Studies have shown that the variant aniridia phenotype is mostly caused by missense mutations, which cause the production of the underlying genes. These underlying genes can exert some of the functions of the *PAX6* gene and affect the development of the eyeball. The present study found that these mutations was mostly concentrated in exon 5-exon 7 of a highly conserved PD area. In this family, three of four affected members in the family had cataract. In addition to aniridia, one family member had ptosis and glaucoma, while another family member had corneal opacity. Furthermore, the heterozygous deletion mutation c.435_445delTAGCGAAAAGC was detected in all four affected members, while this was absent in all unaffected members. This shows that the mutation co-segregates with aniridia in Family 12,002. In aniridia cases, approximately 50–85% had progressive cataract, while 6–75% had glaucoma [16]. The reason why cataract occurs with aniridia remains unknown. The investigators assumed that the dysplasia of the iris may have allowed more light to pass through the crystalline lens that protects the retina, causing the clouding of the lens capsule to occur. However, this remains to be proven.

The *PAX6* gene spans for approximately 23 Kb on chromosome 11p13, contains 14 exons, and encodes a protein of 422 amino acids, which belongs to a highly conserved family of transcription factors. The PAX6 protein has two DNA-binding domains, the paired domain (PD) and homeobox domain (HD), and a linker region connects these two kinds of domains [14]. In its C-terminal end, the proline-serine-threonine-rich (PST) activation domain regulates the expression of *PAX6* downstream genes via phosphorylation [17]. The heterozygous deletion in the *PAX6* gene (c.435_445delTAGCGAAAAGC) identified in the present study was located in exon 7, and was predicted in the protein level to cause the replacement of the serine codon at position 150aa by a threonine codon and a putative stop codon 9 amino acid downstream (p.Ser146ThrfsX9). Furthermore, the mutation located in exon 7 encodes the linker region between PD and HD, and forms a premature termination codon (PTC). One possibility that PTC may bring about is that the mutant *PAX6* may produce a truncated protein with the absence of the HD and PST domain, which is considered functionally inactive. Another possibility is that it may cause nonsense-mediated mRNA decay (NMD), and both of which may finally result in PAX6 underdosage [18–22].

## Conclusion

In conclusion, the phenotype of aniridia patients in a four-generation family was analyzed, and a novel deletion in *PAX6* (c.435_445delTAGCGAAAAGC) was identified as the cause of aniridia in this family. This mutation induced the production of truncated proteins and the generation of null alleles, which resulted in a single-dose deficiency, leading to the absence of the iris. This expands the mutation spectrum of the *PAX6* gene and its phenotype. The present study may be helpful in the genetic diagnosis of aniridia.

## Abbreviations

dbSNP: Database of single nucleotide polymorphisms
HD: Homeobox domain
IRB: Institutional review board
NMD: Nonsense-mediated mRNA decay.
PAX6: Paired box-6
PCR: Polymerase chain reaction
PD: Paired domain
PST: Proline-serine-threonine-rich
PTC: Premature termination codon
WAGR: Wilms tumor, aniridia, genitourinary disorders, and mental Retardation
